# Antistress and antioxidant effects of virgin coconut oil *in vivo*

**DOI:** 10.3892/etm.2014.2045

**Published:** 2014-11-03

**Authors:** SWEE KEONG YEAP, BOON KEE BEH, NORLAILY MOHD ALI, HAMIDAH MOHD YUSOF, WAN YONG HO, SOO PENG KOH, NOORJAHAN BANU ALITHEEN, KAMARIAH LONG

**Affiliations:** 1 Institute of Bioscience, Universiti Putra Malaysia, Serdang, Selangor 43400, Malaysia; 2 Department of Bioprocess Technology, Faculty of Biotechnology and Biomolecular Science, Universiti Putra Malaysia, Serdang, Selangor 43400, Malaysia; 3 Department of Cell and Molecular Biology, Faculty of Biotechnology and Biomolecular Science, Universiti Putra Malaysia, Serdang, Selangor 43400, Malaysia; 4 School of Biomedical Sciences, The University of Nottingham Malaysia Campus, Jalan Broga, Semenyih, Selangor 43500, Malaysia; 5 Biotechnology Research Centre, Malaysian Agricultural Research and Development Institute, Serdang, Selangor 43400, Malaysia

**Keywords:** antioxidant, depression, medium-chain fatty acids

## Abstract

Virgin coconut oil (VCO) has been consumed worldwide for various health-related reasons and some of its benefits have been scientifically evaluated. Medium-chain fatty acids were found to be a potential antidepressant functional food; however, this effect had not been evaluated in VCO, which is rich in polyphenols and medium-chain fatty acids. The aim of this study was to evaluate the antistress and antioxidant effects of VCO *in vivo*, using mice with stress-induced injury. The antistress effect of VCO (administered *per os*, at a dose of 10 ml/kg body weight) was evaluated using the forced swim test and chronic cold restraint stress models. VCO was able to reduce immobility time and restore oxidative stress in mice post-swim test. Furthermore, mice treated with VCO were found to exhibit higher levels of brain antioxidants, lower levels of brain 5-hydroxytryptamine and reduced weight of the adrenal glands. Consequently, the serum cholesterol, triglyceride, glucose and corticosterone levels were also lower in VCO-treated mice. These results suggest the potential value of VCO as an antistress functional oil.

## Introduction

Living in a modern society is often associated with more stressful social conditions (1). Stress is a feedback survival response that strengthens the physical and mental status of an individual. However, extreme stress may compromise mental and somatic health and lead to depression, immunosuppression, hypertension and endocrine disorders (2). This condition has increased the number of patients who suffer from depression, affecting their quality of life and, subsequently, the socio-economic balance (1). Various drugs are currently used to manage stress and depression, including diazepam, caffeine and certain anabolic steroids. However, these drugs may be associated with severe toxicity and side effects (2). Thus, stress management through dietary modifications (1) and natural herbs (2) may be a valuable alternative to antidepressant drugs.

Virgin coconut oil (VCO), which may be produced from fresh coconut meat, coconut milk or coconut milk residue, is rich in medium-chain triglycerides (TGs) and lauric acid (3). VCO has been widely consumed as a health food and has also been utilised for cosmeceutical purposes (3). Previous studies reported the benefits of VCO consumption, including antiulcerogenic, antinociceptive, anti-inflammatory (4), antihypercholesterolemic (5), antimicrobial and hepatoprotective (6) effects. Nutrition and antioxidant supplements have been considered to be beneficial for the recovery from exercise-induced oxidative stress (7). Shinohara *et al* (1) demonstrated the potential of a medium-chain fatty acid-containing dietary oil, which was found to be effective as an antidepressant in the forced swim test. As VCO is rich in medium-chain fatty acids (3) and polyphenols (4), it may be used as an antistress and antidepressant nutritional oil. Thus, this study aimed to investigate the antistress effect of VCO using the forced swim test chronic restraint stress models.

## Materials and methods

### VCO samples

The VCO samples for this study were produced via dry processes according to the method reported by Kamariah *et al* (3). The medium-chain fatty acid (C6–C12) content of this VCO sample was >64% of its saturated fatty acid content (8).

### Animals

A total of 56 male inbred BALB/c mice, aged 6 weeks and weighing ~25 g, were purchased from the Comparative Medicine And Technology Unit, Institute of Bioscience, Universiti Putra Malaysia (Serdang, Malaysia) and acclimatized for 2 weeks under controlled conditions at ~22°C and 55% humidity, with 12-h day/dark light cycles. The animals had access to standard pellets (Gold Coin, Kuala Lumpur, Malaysia) and distilled water *ad libitum*. This study was conducted according to the guidelines for the care and use of laboratory animals of the Animal Care and Use Committee, Universiti Putra Malaysia.

### Forced swim test

A total of 24 mice were randomly divided into three groups (n=8 per group), namely the untreated control, positive control and VCO groups, which were administered 250 μl saline, 2 mg/kg body weight (bw) diazepam and 10 ml/kg bw VCO, respectively, *per os* (p.o.) for a total of 7 days. On day 6, all the mice were allowed to swim individually for 6 min for adaptation. On day 7, the mice were allowed to swim individually for 6 min and the duration of immobility (period during which the mice only floated in the upright position with minimum movement to keep their heads above water) was scored 3 min after placement into the water (1). Immediately following the swim test, the mice were sacrificed via isoflurane and their livers were harvested and homogenized. The liver homogenates were subjected to superoxide dismutase (SOD) and malondialdehyde (MDA) determination according to the method described by Ho *et al* (8).

### Chronic cold restraint stress test

A total of 32 mice were randomly divided into four groups (n=8 per group), namely the no-stress control and the untreated control, positive control and VCO stress groups, which were administered 250 μl saline, 2 mg/kg bw diazepam and 10 ml/kg bw VCO, respectively, p.o. for a total of 28 days. From day 21 onward, the mice in the untreated control, positive control and VCO stress groups were daily subjected to 1 h of cold restraint stress at 4°C for a total of 7 days. At the end of the experiment, the mice were sacrificed via isoflurane and their sera were collected and subjected to total cholesterol, TG, total protein, glucose (all kits from BioVision, Mountain View, CA, USA) and corticosterone (Cayman Chemical Company, Ann Arbor, MI, USA) determination, according to the manufacturer’s instructions. The adrenal glands were also removed and weighed. The mouse brains were frozen in liquid nitrogen and homogenized in a mixture of HCl (0.01N) and butanol. Dopamine (DA) and serotonin (IBL International GmbH, Hamburg, Germany) from the aqueous phase of the acid extract were quantified using ELISA (2), while brain SOD and MDA were determined according to method described by Ho *et al* (8).

### Statistical analysis

All the results are presented as means ± standard error of the mean. Statistical significance was analysed using one-way analysis of variance with the Duncan test as post-hoc analysis by SPSS 18.0 software (SPSS, Inc., Chicago, IL, USA). P<0.05 was considered to indicate a statistically significant difference.

## Results

### Effect of VCO on swimming endurance ability

**Figure 1:**
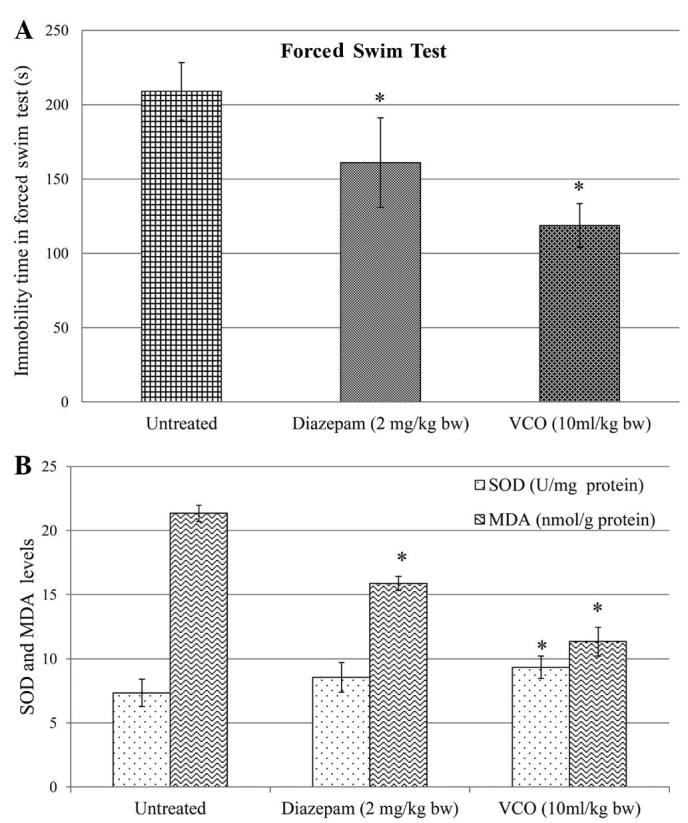
Antistress effect of VCO during the forced swim test. (A) Mice fed with 10 ml/kg body weight VCO exhibited reduced immobility time during the forced swim test; (B) Serum SOD and MDA levels post-forced swim test. ^*^Significant difference (P<0.05) compared to the untreated controls. VCO, virgin coconut oil; SOD, superoxide dismutase; MDA, malondialdehyde.

The mice were subjected to forced swim stress and the results on the time of immobility are shown in Fig. 1. VCO and diazepam were able to significantly reduce the time of immobility compared to the untreated control mice (Fig. 1A). The serum antioxidant SOD enzyme level was increased and lipid peroxidation was significantly reduced in VCO-treated mice post-forced swim test (Fig. 1B).

### Effect of VCO on chronic cold restraint stress

The mice exposed to 7 days of chronic cold restraint stress exhibited significant changes in their serum biochemical profiles and brain monoamine and oxidation levels. Compared to the normal no-stress control group, the untreated stress control group mice were found to exhibit higher serum cholesterol, TG, glucose and corticosterone levels, associated with increased adrenal gland weight. Higher 5-hydroxytryptamine (5-HT) and MDA and lower SOD levels were detected in the brain homogenates. However, the levels of DA were not significantly altered. Treatment with diazepam and VCO were able to restore the serum biochemical profile and reduce the weight of the adrenal glands. Furthermore, the oxidation and 5-HT levels, which were higher in the untreated stress control group mice, were significantly reduced in the diazepam- and VCO-treated mice.

## Discussion

In this study, the effects of VCO on exercise-induced and chronic cold restraint stress were evaluated. Stress and depression are major contributors to psychiatric pathologies and have been found to alter the neurotransmitter and biochemical profiles and the oxidation status in the central nervous system (9).

A longer immobility time was considered as an indicator of stress and depression in untreated mice undergoing the forced swim test (1). The mice treated with VCO and diazepam exhibited a shorter immobility time compared to untreated mice. This effect may be attributed to the high medium-chain fatty acid content of VCO. Shinohara *et al* (1) reported that a daily intake of medium-chain fatty acids may help prevent the development of stress-induced depression in mice using a similar forced swim test model.

In addition to exercise-induced stress, the antistress effect of VCO was also evaluated using a chronic cold restraint stress model. Chronic stress was found to increase adrenal gland weight and serum corticosterone and 5-HT levels (10). The increase in adrenal hormones and release of corticosterone are known to induce hyperinsulinemia and insulin resistance, thus elevating the serum glucose, cholesterol and TG levels (11). However, prolonged chronic stress was found to be associated with reduced DA levels, indicating reduction of motivation to food reward and depression (12). Compared to the no-stress control group, the untreated-stress control mice in the present study were found to exhibit alterations in their serum biochemical profiles (higher total cholesterol, TG, glucose and corticosterone levels), increased adrenal weight and increased brain 5-HT levels. Furthermore, the reduction of the brain DA level may indicate depression and lower motivation in the mice (Table I). Treatment with VCO and diazepam were able to restore the serum biochemical profiles and brain 5-HT levels and significantly reduced adrenal gland weight. However, only diazepam was able to significantly restore the level of DA in this study.

Oxidation and generation of free radicals commonly occur due to the high consumption of oxygen during exercise. The balance between oxidants and antioxidants is commonly measured using the forced swim test (7). In addition to exercise-induced stress, chronic stress has also been found to induce oxidation, leading to neuronal cell damage and death (9). In this study, untreated mice undergoing the forced swim test (Fig. 1B) and chronic cold restraint stress (Table I) were found to exhibit higher lipid peroxidation (MDA) and lower antioxidant enzyme SOD levels. VCO was able to reduce lipid peroxidation and increase the activity of SOD in the serum of mice undergoing the forced swim test and the brains of mice subjected to chronic cold restraint. It was previously reported that VCO is rich in polyphenols and these antioxidants may contribute to the increased levels of antioxidant enzymes, which subsequently reduce lipid peroxidation and inflammation in VCO-treated mice (4). Restoration of antioxidant levels in the brain may help prevent further neuronal damage and avoid subsequent depletion of monoamines, including DA (9). In conclusion, the present study demonstrated the potential of VCO in preventing exercise- and chronic cold restraint stress-induced damage and restoring the antioxidant balance. This promising antistress activity may be attributed to the polyphenols and medium-chain fatty acids present in VCO.

## Figures and Tables

**Table I:** Antistress effect of VCO on the serum biochemical profiles, adrenal glands and brains of mice subjected to the chronic cold restraint test.

	Serum				Adrenal gland		Brain		
			
Groups	Cholesterol (mmol/l)	TG (mmol/l)	Glucose (mmol/l)	Corticosterone (μg/100 ml)	Weight (mg/100 g)	5-HT (μg/g)	Dopamine (μg/g)	SOD (U/mg protein)	MDA (nmol/g protein)
No-stress control	4.97±0.34^a^	2.69±0.47^a^	6.13±0.77^a^	7.32±0.74	32.35±1.25^a^	0.72±0.04^a^	0.58±0.09^a^	12.57±1.32^a^	3.51±0.88^a^
Untreated stress control	5.83±0.41	3.77±0.46	9.31±0.48	17.88±1.14	36.77±1.32	1.81±0.06	0.51±0.02	5.97±1.77	16.82±1.76
Diazapem (2 mg/kg)^b^	4.86±0.51^a^	2.81±0.33^a^	5.91±0.26^a^	7.84±0.93^a^	31.66±0.98^a^	0.81±0.04^a^	0.58±0.03^a^	8.83±0.79^a^	7.44±1.11^a^
VCO (10 ml/kg)	4.96±0.32^a^	3.01±0.25^a^	6.20±0.60^a^	8.23±0.95^a^	33.10±0.96^a^	0.70±0.03^a^	0.55±0.04	9.85±1.26^a^	5.38±1.59^a^

a Significant difference (P<0.05) compared to the untreated stress control group.

b Positive stress control group.

Data are presented as means ± standard error of the mean. VCO, virgin coconut oil; TG, triglyceride; 5-HT, 5-hydroxytryptamine; SOD, superoxide dismutase; MDA, malondialdehyde.
